# Crystal structure of [1,1′′′-bis­(pyrimidin-2-yl)-4,4′:2′,2′′:4′′,4′′′-quaterpyridine-1,1′′′-diium-κ^2^ *N* ^1′^,*N* ^1′′^]bis­[2-(pyridin-2-yl)phenyl-κ^2^ *N*,*C* ^1^]iridium(III) tris­(hexa­fluorido­phosphate) aceto­nitrile tris­olvate

**DOI:** 10.1107/S2056989015012463

**Published:** 2015-07-04

**Authors:** Benjamin J. Coe, Martyn K. Peers, James Raftery, Nigel S. Scrutton

**Affiliations:** a School of Chemistry, University of Manchester, Manchester M13 9PL, England; b Manchester Institute of Biotechnology, Faculty of Life Sciences, University of Manchester, 131 Princess Street, Manchester M1 7DN, England

**Keywords:** crystal structure, iridium(III), cyclo­metalated, 4,4′:2′,2′′:4′′,4′′′-quaterpyridyl ligand

## Abstract

In the title compound, [Ir(C_11_H_8_N)_2_(C_28_H_20_N_8_)](PF_6_)_3_·3CH_3_CN or [Ir^III^(ppy)_2_{(2-pym)_2_qpy^2+^}](PF_6_)_3_·3CH_3_CN (ppy = deprotonated 2-phenyl­pyridine, pym = pyrimidyl and qpy = 4,4′:2′,2′′:4′′,4′′′-quaterpyrid­yl), the Ir^3+^ cation is coordinated by two C atoms and four N atoms in a slightly distorted octa­hedral geometry. The asymmetric unit consists of one complex trication, three hexa­fluorido­phosphate anions and three aceto­nitrile solvent mol­ecules. The average Ir—C distance is 2.011 (14) Å, the average Ir—N(ppy) distance is 2.05 (6) Å and the average Ir—N(qpy) distance is longer at 2.132 (10) Å. The dihedral angles within the 4,4′-bipyridyl units are 31.5 (6) and 23.8 (7)°, while those between the 2-pym and attached pyridyl rings are rather smaller, at 11.7 (9) and 7.1 (9)°. The title compound was refined as a two-component inversion twin.

## Abstract 2

In the title compound, the Ir^3+^ cation is coordinated by two C atoms and four N atoms in a slightly distorted octa­hedral geometry. The asymmetric unit consists of one complex trication, three hexa­fluorido­phosphate anions and three aceto­nitrile solvent mol­ecules.

## Chemical context   

Iridium complexes of cyclo­metalating ligands have been studied widely, mainly due to their inter­esting photophysical properties (Flamigni *et al.*, 2007 ▸; You & Nam, 2012 ▸; Ladouceur & Zysman-Colman, 2013 ▸). Complexes of the form [Ir^III^(ppy)_2_(N–N)]^+^ (N–N = 2,2′-bipyridyl or a related α-di­imine ligand) are well known, and many examples have been structurally characterized (*e.g*. Ladouceur *et al.*, 2010 ▸; Zhao *et al.*, 2010 ▸; Constable *et al.*, 2013 ▸; Schneider *et al.*, 2014 ▸).
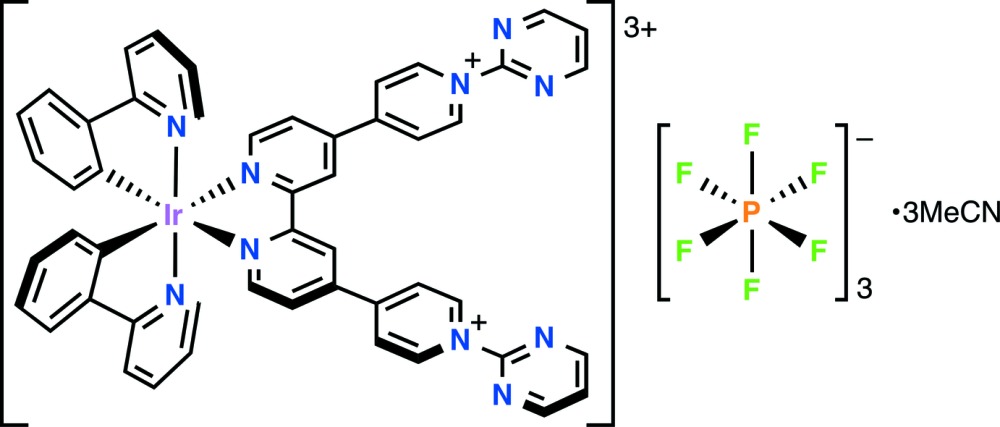


However, such compounds containing ligands with pyridin­ium substituents are scarce, and the only ones reported to our knowledge are the complex salts [Ir^III^(C–N)_2_(Me_2_qpy^2+^)][PF_6_]_3_ (L–L = ppy or benzo[*h*]quinoline) (Ahmad *et al.*, 2014 ▸). We report here a related new compound and what appears to be the first X-ray crystal structure determination of an iridium complex containing a qpy-based ligand.

## Structural commentary   

**Figure 1:**
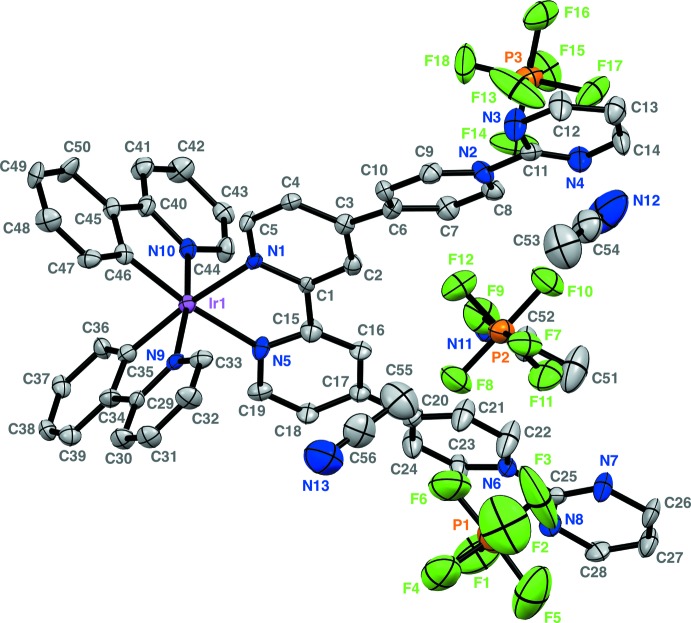
View of the molecular components of (I) (50% probability displacement ellipsoids)

The mol­ecular structure (Fig. 1 ▸) of the complex cation in [Ir^III^(ppy)_2_{(2-pym)_2_qpy^2+^}][PF_6_]_3_·3CH_3_CN (I) is as indicated by ^1^H NMR spectroscopy, with a slightly distorted octa­hedral coordination geometry. The bite angle of the qpy-based ligand is 76.6 (2)°, while those of the ppy ligands are slightly larger at 80.1 (6) and 80.8 (5)°. As for other related complexes (Ladouceur *et al.*, 2010 ▸; Zhao *et al.*, 2010 ▸; Constable *et al.*, 2013 ▸; Schneider *et al.*, 2014 ▸), the strong *trans* effects of a σ-bonded phenyl ring (Coe & Glenwright, 2000 ▸) causes these units to adopt a *cis* orientation, so that the pyridyl rings of the ppy ligands are oriented *trans*. The structural *trans* effect of the phenyl rings is shown by the *ca* 0.08 Å lengthening of the Ir—N(qpy) distances [average = 2.132 (10) Å] with respect to the Ir—N(ppy) ones [average = 2.05 (6) Å]. The Ir—C distances (Table 1 ▸) are shorter still, with an average value of 2.01 (14) Å. All of the geometric parameters around the Ir^3+^ cation are similar to those reported for related structures.

The dihedral angles within the 4,4′-bipyridyl units in (I) are larger than those [20.8 (6) and 21.0 (5)°] in the only other structurally characterized complex of the (2-pym)_2_qpy^2+^ ligand, [Ru^II^(bpy)_2_{(2-pym)_2_qpy^2+^}][PF_6_]_4_ (bpy = 2,2′-bi­pyr­id­yl) (Coe *et al.*, 2011 ▸). On the other hand, the dihedral angles between the 2-pyrimidyl and attached pyridyl rings are closely similar in (I), whereas two quite different such angles are observed in [Ru^II^(bpy)_2_{(2-pym)_2_qpy^2+^}][PF_6_]_4_ [6.0 (9) and 20.0 (5)°].

## Supra­molecular features   

**Figure 2:**
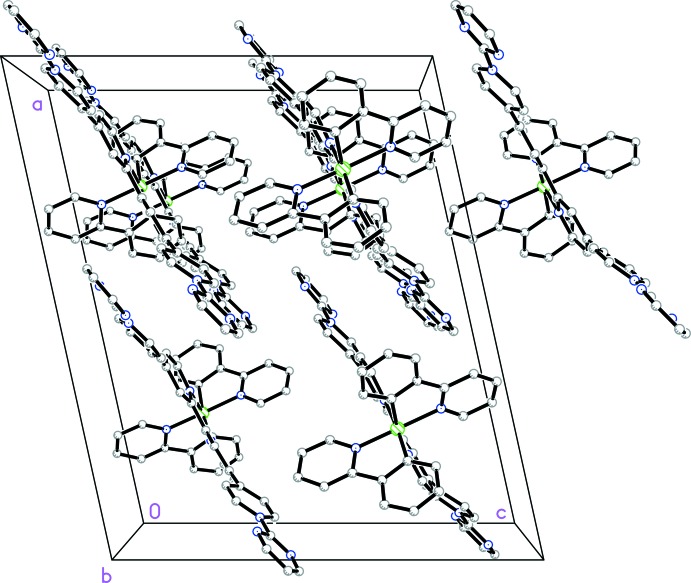
Crystal packing diagram, viewed approximately along the *b* axis, showing the alignment of the qpy fragments. The H atoms, PF_6_^−^ anions and aceto­nitrile solvent mol­ecules have been removed for clarity.

**Figure 3:**
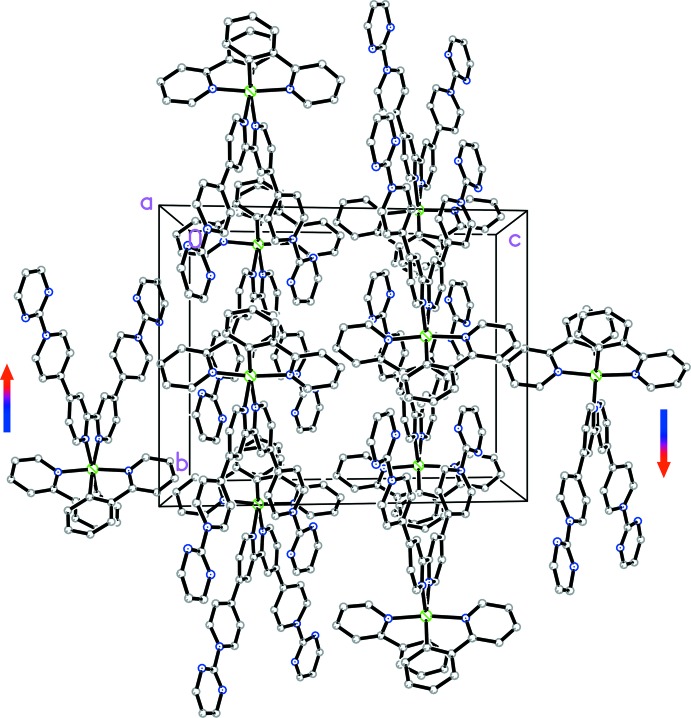
Crystal packing diagram, viewed approximately along the *a* axis, showing the anti­parallel alignment of the mol­ecular dipoles (represented by arrows for the extreme left and right complexes). The H atoms, PF_6_^−^ anions and aceto­nitrile solvent mol­ecules have been removed for clarity.

The unit cell contains four complex cations with their qpy units aligned approximately parallel (Fig. 2 ▸). There may be a weak π-stacking inter­action between a 2-pym ring and one of the rings of the bpy fragment in an adjacent complex, with a centroid-to-centroid distance of 3.854 (8) Å and a dihedral angle of 9.8 (6)°. Ru^II^ complexes of (2-pym)_2_qpy^2+^ and related ligands show inter­esting non-linear optical (NLO) properties (Coe *et al.* 2005 ▸). In this context, crystal packing arrangements are of great importance because macroscopic polarity is necessary for the existence of bulk quadratic NLO effects. The space group *Cc* adopted by (I) is non-centrosymmetric, potentially affording a polar material that could display such NLO properties. However, the overall orientation of the dipoles formed by the electron-donating Ir^III^(ppy)_2_ units and the accepting (2-pym)_2_qpy^2+^ ligands is anti­parallel (Fig. 3 ▸). Therefore, significant bulk quadratic NLO behaviour is not expected for this particular crystal form.

## Synthesis and crystallization   

The new compound (I) was synthesised simply by cleaving the commercial chloride-bridged dimer [Ir^III^(ppy)_2_Cl]_2_ with the proligand salt [(2-pym)_2_qpy^2+^]Cl_2_ (Coe *et al.*, 2011 ▸) in refluxing 2-meth­oxy­ethanol/water.

[Ir^III^(ppy)_2_Cl]_2_ (40 mg, 0.037 mmol) and *N*′′,*N*′′′-di(2-pyrimid­yl)-4,4′:2′,2′′:4′′,4′′′-quaterpyridinium chloride·2.3H_2_O (47 mg, 0.081 mmol) in argon-sparged 2-meth­oxy­ethanol/water (3:1, 10 ml) were heated at reflux for 20 h. After cooling to room temperature, the solvent was removed by rotary evaporation and the residue redissolved in a minimum volume of methanol to which was added an excess of solid NH_4_PF_6_. Cold water was added and the precipitate was filtered off and washed with water. The product was purified by column chromatography on silica gel, eluting with 0.1 *M* NH_4_PF_6_ in aceto­nitrile, to afford a brown–green solid. Yield: 68 mg (65%). Analysis calculated for C_50_H_36_F_18_IrN_10_P_3_·H_2_O: C 42.2, H 2.7, N 9.8%; found: C 42.0, H 2.5, N 9.6%. Spectroscopic analysis: ^1^H NMR (400 MHz, CD_3_CN, δ, p.p.m.) 10.12 (4H, *dd*, *J* = 7.5, 1.9 Hz), 9.18 (2H, *d*, *J* = 1.4 Hz), 9.13 (4H, *d*, *J* = 4.9 Hz), 8.68 (4H, *dd*, *J* = 7.4, 1.8 Hz), 8.31 (2H, *d*, *J* = 5.6 Hz), 8.13 (2H, *dt*, *J* = 8.1, 0.8 Hz), 8.05 (2H, *dd*, *J* = 5.7, 1.8 Hz), 7.93–7.87 (8H), 7.71 (2H, *ddd*, *J* = 5.9, 1.5, 0.7 Hz), 7.14–6.98 (8H), 6.33 (2H, *dd*, *J* = 7.6, 0.9 Hz). MALDI–MS *m*/z = 1405 ({*M*}^+^), 1260 ({*M* – PF_6_}^+^), 1115 ({*M* – 2PF_6_}^+^), 970 ({*M* – 3PF_6_}^+^).

Single crystals (amber plates) suitable for X-ray diffraction studies were grown by slow diffusion of diethyl ether vapour into an aceto­nitrile solution at room temperature.

## Other Characterization   

The complex salt (I) shows a relatively weak, broad visible absorption band at λ_max_ = 562 nm (∊ = 1,800 *M* ^−1^ dm^3^) in aceto­nitrile. Based on the results of time-dependent density functional theory (TD–DFT) calculations on the related complex [Ir^III^(ppy)_2_(Me_2_qpy^2+^)]^3+^ (Peers, 2012 ▸), this absorption is attributable to d→π* metal-to-ligand charge-transfer (MLCT) transitions directed towards the qpy-based ligand, with significant contributions by the ppy ligands to the donor orbitals introducing also ligand-to-ligand CT character. Below 500 nm, absorption increases steadily into the UV region, with another maximum at 378 nm (∊ = 16,600 *M* ^−1^ dm^3^), and a shoulder at *ca* 410 nm. By way of contrast, the lowest energy band for [Ir^III^(ppy)_2_(Me_2_qpy^2+^)][PF_6_]_3_ appears at λ_max_ = 531 nm (∊ = 1,200 *M* ^−1^ dm^3^) in aceto­nitrile (Peers, 2012 ▸). The substantial red-shift of this band on moving to (I) is due to the enhanced electron-accepting ability of the *N*-(2-pyrimid­yl)pyridinium groups. The higher intensity for (I) is a consequence of extended π-conjugation involving the 2-pym rings.

Cyclic voltammetric studies on (I) reveal an irreversible oxidation process at *E*_pa_ = 1.43 V *vs* Ag–AgCl {aceto­nitrile, 0.1 *M* [*N*(*n*-Bu_4_)]PF_6_, 2 mm Pt disc working electrode, 100 mV s^−1^, ferrocene/ferrocenium standard at 0.44 V (Δ*E*_p_ = 70–90 mV)}. The reductive region shows a reversible wave at *E*_1/2_ = −0.29 V (Δ*E*_p_ = 80 mV), followed by an irreversible process with *E*_pc_ = −0.79 V. Based on the relative peak currents, the reversible wave is assigned as a two-electron process involving reduction of both pyridinium units. The redox behaviour of this complex can be rationalized with the aid of DFT results obtained for [Ir^III^(ppy)_2_(Me_2_qpy^2+^)]^3+^ (Peers, 2012 ▸). The irreversible oxidation wave corresponds with removing an electron from the HOMO comprising the Ir and ppy ligands. The first and second reductions involve adding electrons to the LUMO based on the (2-pym)_2_qpy^2+^ ligand. The oxidation occurs at the same *E*_pa_ value for (I) and its methyl­ated analogue, [Ir^III^(ppy)_2_(Me_2_qpy^2+^)][PF_6_]_3_, but the first two reductions appear as overlapping reversible waves at *E*_1/2_ = −0.62 V (Δ*E*_p_ = 70 mV) and *E*_1/2_ = −0.73 V (Δ*E*_p_ = 60 mV) in the latter compound. These waves can be resolved by using differential pulse voltammetry (potential increment = 2 mV, amplitude = 50 mV, pulse width = 0.01 s). The anodic shift in the reduction waves is consistent with the qpy-based ligand being more electron-deficient, and therefore easier to reduce, in (I). The lack of splitting of these waves in (I) indicates that electronic communication between the pyridyl radicals is diminished with respect to its methyl analogue. Inter­estingly, for the related compound [Ru^II^(bpy)_2_{(2-pym)_2_qpy^2+^}][PF_6_]_4_, the first two reductions are irreversible under the same conditions using a glassy carbon working electrode (Coe *et al.*, 2011 ▸).

## Refinement   

The structure was solved by direct methods. The two rings of one of the ppy ligands are indistinguishable by bond lengths, and the presented structure gives the lowest *R* factors. Crystal twinning is present. There is a pseudo-twofold axis that manifests itself as high correlation between parameters during refinement. The non-hydrogen atoms were refined anisotropically, but a rigid bond restraint (RIGU in *SHELX*) was applied for atoms with pseudo-symmetry-related counterparts. H atoms were included in calculated positions with C—H bond lengths of 0.95 (CH), 0.99 (CH_2_) and 0.98 (CH_3_) Å; *U*_ĩso_(H) values were fixed at 1.2*U*_eq_(C) except for CH_3_ where it was 1.5*U*_eq_(C). Crystal data, data collection and structure refinement details are given in Table 2 ▸.

## Supplementary Material

Crystal structure: contains datablock(s) I. DOI: 10.1107/S2056989015012463/zl2625sup1.cif

Structure factors: contains datablock(s) I. DOI: 10.1107/S2056989015012463/zl2625Isup2.hkl

CCDC reference: 1409461

Additional supporting information:  crystallographic information; 3D view; checkCIF report

## Figures and Tables

**Table 1:** Selected geometric parameters (, )

C35Ir1	2.021(16)	Ir1N9	2.095(13)
C46Ir1	2.000(15)	Ir1N1	2.125(11)
Ir1N10	2.011(14)	Ir1N5	2.139(11)
			
C46Ir1N10	80.8(5)	C35Ir1N1	172.7(6)
C46Ir1C35	86.0(3)	N9Ir1N1	93.8(4)
N10Ir1C35	94.2(6)	C46Ir1N5	173.1(6)
C46Ir1N9	93.9(5)	N10Ir1N5	94.4(5)
N10Ir1N9	172.5(2)	C35Ir1N5	99.4(6)
C35Ir1N9	80.1(6)	N9Ir1N5	91.3(4)
C46Ir1N1	98.4(6)	N1Ir1N5	76.6(2)
N10Ir1N1	92.3(5)		

**Table 2:** Experimental details

Crystal data	
Chemical formula	[Ir(C_11_H_8_N)_2_(C_28_H_20_N_8_)](PF_6_)_3_3C_2_H_3_N
*M*_r_	1527.16
Crystal system, space group	Monoclinic, *C* *c*
Temperature (K)	100
*a*, *b*, *c* ()	22.2647(16), 14.6139(11), 18.6288(14)
()	102.447(1)
*V* (^3^)	5918.9(8)
*Z*	4
Radiation type	Mo *K*
(mm^1^)	2.45
Crystal size (mm)	0.25 0.20 0.03

Data collection	
Diffractometer	Bruker SMART CCD area detector
Absorption correction	Multi-scan (*SADABS*; Bruker, 2001 ▸)
*T*_min_, *T*_max_	0.697, 0.930
No. of measured, independent and observed [*I* > 2(*I*)] reflections	25080, 13146, 11295
*R*_int_	0.037
(sin /)_max_ (^1^)	0.669

Refinement	
*R*[*F* ^2^ > 2(*F* ^2^)], *wR*(*F* ^2^), *S*	0.041, 0.093, 1.00
No. of reflections	13146
No. of parameters	824
No. of restraints	434
H-atom treatment	H-atom parameters constrained
_max_, _min_ (e ^3^)	1.24, 1.00
Absolute structure	Refined as an inversion twin
Absolute structure parameter	0.384(7)

Computer programs: *SMART* and *SAINT-Plus* (Bruker, 2003 ▸), *SHELXTL* (Sheldrick, 2008 ▸) and *SHELXL2014*/7 (Sheldrick, 2015 ▸).

## supplementary crystallographic information

### Crystal data

**Table:**

[Ir(C_11_H_8_N)_2_(C_28_H_20_N_8_)](PF_6_)_3_·3C_2_H_3_N	*F*(000) = 3024
*M**_r_* = 1527.16	*D*_x_ = 1.714 Mg m^−^^3^
Monoclinic, *C**c*	Mo *K*α radiation, λ = 0.71073 Å
*a* = 22.2647 (16) Å	Cell parameters from 5333 reflections
*b* = 14.6139 (11) Å	θ = 2.2–23.6°
*c* = 18.6288 (14) Å	µ = 2.44 mm^−^^1^
β = 102.447 (1)°	*T* = 100 K
*V* = 5918.9 (8) Å^3^	Plate, brown
*Z* = 4	0.25 × 0.20 × 0.03 mm

### Data collection

**Table:**

Bruker SMART CCD area-detector diffractometer	11295 reflections with *I* > 2σ(*I*)
φ and ω scans	*R*_int_ = 0.037
Absorption correction: multi-scan (*SADABS*; Bruker, 2001)	θ_max_ = 28.4°, θ_min_ = 1.7°
*T*_min_ = 0.697, *T*_max_ = 0.930	*h* = −29→29
25080 measured reflections	*k* = −19→19
13146 independent reflections	*l* = −24→24

### Refinement

**Table:**

Refinement on *F*^2^	Hydrogen site location: inferred from neighbouring sites
Least-squares matrix: full	H-atom parameters constrained
*R*[*F*^2^ > 2σ(*F*^2^)] = 0.041	*w* = 1/[σ^2^(*F*_o_^2^) + (0.0493*P*)^2^] where *P* = (*F*_o_^2^ + 2*F*_c_^2^)/3
*wR*(*F*^2^) = 0.093	(Δ/σ)_max_ < 0.001
*S* = 1.00	Δρ_max_ = 1.24 e Å^−^^3^
13146 reflections	Δρ_min_ = −1.00 e Å^−^^3^
824 parameters	Absolute structure: Refined as an inversion twin.
434 restraints	Absolute structure parameter: 0.384 (7)

### Special details

**Table:**

Geometry. All esds (except the esd in the dihedral angle between two l.s. planes) are estimated using the full covariance matrix. The cell esds are taken into account individually in the estimation of esds in distances, angles and torsion angles; correlations between esds in cell parameters are only used when they are defined by crystal symmetry. An approximate (isotropic) treatment of cell esds is used for estimating esds involving l.s. planes.
Refinement. Refined as a 2-component inversion twin.

### Fractional atomic coordinates and isotropic or equivalent isotropic displacement parameters (Å^2^)

**Table:**

	*x*	*y*	*z*	*U*_iso_*/*U*_eq_	
C1	0.2259 (6)	0.2672 (9)	0.2388 (7)	0.016 (2)	
C2	0.1948 (6)	0.3457 (9)	0.2574 (7)	0.022 (3)	
H2	0.2117	0.4048	0.2540	0.027*	
C3	0.1400 (6)	0.3367 (9)	0.2803 (7)	0.022 (3)	
C4	0.1148 (6)	0.2499 (9)	0.2826 (7)	0.021 (3)	
H4	0.0773	0.2415	0.2982	0.025*	
C5	0.1461 (6)	0.1756 (9)	0.2613 (8)	0.022 (3)	
H5	0.1283	0.1165	0.2612	0.027*	
C6	0.1124 (8)	0.4180 (9)	0.3043 (10)	0.025 (3)	
C7	0.1177 (6)	0.5038 (10)	0.2731 (7)	0.029 (3)	
H7	0.1422	0.5094	0.2374	0.035*	
C8	0.0887 (6)	0.5808 (9)	0.2923 (8)	0.028 (3)	
H8	0.0926	0.6382	0.2699	0.033*	
C9	0.0490 (6)	0.4917 (9)	0.3774 (8)	0.029 (3)	
H9	0.0257	0.4879	0.4144	0.034*	
C10	0.0776 (6)	0.4132 (9)	0.3577 (8)	0.022 (2)	
H10	0.0732	0.3564	0.3809	0.027*	
C11	0.0220 (6)	0.6533 (9)	0.3638 (7)	0.024 (3)	
C12	−0.0487 (6)	0.7102 (9)	0.4174 (8)	0.045 (4)	
H12	−0.0782	0.7039	0.4471	0.055*	
C13	−0.0393 (7)	0.7960 (10)	0.3933 (8)	0.039 (3)	
H13	−0.0618	0.8481	0.4030	0.047*	
C14	0.0060 (7)	0.7995 (10)	0.3538 (9)	0.045 (4)	
H14	0.0163	0.8577	0.3371	0.054*	
C15	0.2866 (7)	0.2711 (10)	0.2175 (9)	0.029 (3)	
C16	0.3129 (6)	0.3483 (9)	0.2000 (7)	0.023 (3)	
H16	0.2934	0.4058	0.2024	0.028*	
C17	0.3691 (6)	0.3434 (10)	0.1782 (8)	0.025 (3)	
C18	0.3968 (6)	0.2579 (10)	0.1791 (8)	0.029 (3)	
H18	0.4362	0.2525	0.1675	0.035*	
C19	0.3678 (6)	0.1829 (10)	0.1964 (7)	0.027 (3)	
H19	0.3869	0.1249	0.1962	0.032*	
C20	0.4021 (8)	0.4263 (8)	0.1574 (11)	0.027 (4)	
C21	0.3913 (7)	0.5118 (11)	0.1809 (10)	0.049 (5)	
H21	0.3612	0.5207	0.2094	0.058*	
C22	0.4228 (9)	0.5822 (11)	0.1639 (12)	0.054 (6)	
H22	0.4137	0.6414	0.1799	0.065*	
C23	0.4759 (6)	0.4923 (9)	0.0972 (8)	0.033 (3)	
H23	0.5045	0.4867	0.0663	0.039*	
C24	0.4456 (8)	0.4188 (10)	0.1122 (10)	0.043 (4)	
H24	0.4531	0.3610	0.0925	0.051*	
C25	0.5026 (5)	0.6526 (9)	0.1111 (7)	0.023 (2)	
C26	0.5170 (7)	0.8075 (10)	0.1180 (8)	0.039 (3)	
H26	0.5070	0.8670	0.1324	0.047*	
C27	0.5625 (7)	0.7970 (10)	0.0824 (9)	0.041 (3)	
H27	0.5830	0.8492	0.0688	0.049*	
C28	0.5797 (5)	0.7123 (9)	0.0654 (8)	0.039 (3)	
H28	0.6146	0.7043	0.0444	0.047*	
C29	0.3416 (6)	0.0041 (8)	0.3583 (7)	0.021 (2)	
C30	0.3697 (6)	−0.0094 (10)	0.4323 (7)	0.029 (3)	
H30	0.4010	−0.0544	0.4455	0.035*	
C31	0.3525 (7)	0.0409 (11)	0.4848 (8)	0.029 (3)	
H31	0.3710	0.0319	0.5352	0.035*	
C32	0.3089 (7)	0.1038 (12)	0.4643 (8)	0.032 (3)	
H32	0.2968	0.1414	0.5002	0.038*	
C33	0.2812 (5)	0.1147 (11)	0.3905 (7)	0.025 (3)	
H33	0.2495	0.1588	0.3764	0.030*	
C34	0.3566 (7)	−0.0476 (10)	0.2978 (8)	0.025 (3)	
C35	0.3202 (7)	−0.0283 (11)	0.2284 (9)	0.024 (3)	
C36	0.3307 (8)	−0.0773 (8)	0.1672 (10)	0.025 (4)	
H36	0.3059	−0.0649	0.1200	0.030*	
C37	0.3771 (6)	−0.1442 (9)	0.1744 (8)	0.025 (3)	
H37	0.3836	−0.1781	0.1332	0.030*	
C38	0.4124 (6)	−0.1580 (9)	0.2432 (7)	0.026 (3)	
H38	0.4454	−0.2006	0.2492	0.032*	
C39	0.4022 (6)	−0.1135 (10)	0.3030 (8)	0.027 (3)	
H39	0.4269	−0.1277	0.3499	0.032*	
C40	0.1739 (6)	−0.0114 (9)	0.1020 (8)	0.025 (3)	
C41	0.1474 (6)	−0.0268 (10)	0.0296 (7)	0.031 (3)	
H41	0.1184	−0.0750	0.0171	0.037*	
C42	0.1623 (7)	0.0272 (11)	−0.0272 (9)	0.036 (3)	
H42	0.1429	0.0162	−0.0771	0.044*	
C43	0.2074 (7)	0.0991 (11)	−0.0083 (8)	0.026 (3)	
H43	0.2188	0.1363	−0.0450	0.031*	
C44	0.2327 (6)	0.1113 (11)	0.0641 (7)	0.029 (3)	
H44	0.2619	0.1591	0.0770	0.034*	
C45	0.1616 (7)	−0.0586 (8)	0.1663 (8)	0.021 (3)	
C46	0.1983 (7)	−0.0308 (10)	0.2348 (8)	0.020 (3)	
C47	0.1897 (7)	−0.0784 (9)	0.2961 (9)	0.026 (4)	
H47	0.2142	−0.0643	0.3432	0.031*	
C48	0.1464 (7)	−0.1454 (10)	0.2891 (8)	0.033 (4)	
H48	0.1405	−0.1745	0.3326	0.040*	
C49	0.1101 (6)	−0.1740 (9)	0.2227 (7)	0.032 (3)	
H49	0.0807	−0.2216	0.2205	0.038*	
C50	0.1188 (6)	−0.1296 (10)	0.1591 (7)	0.033 (3)	
H50	0.0960	−0.1474	0.1120	0.039*	
C51	0.2934 (8)	0.7144 (9)	0.3228 (9)	0.083 (5)	
H51A	0.2700	0.7210	0.3614	0.125*	
H51B	0.2848	0.7665	0.2890	0.125*	
H51C	0.3375	0.7127	0.3452	0.125*	
C52	0.2755 (5)	0.6290 (7)	0.2821 (7)	0.050 (3)	
C53	0.2087 (8)	0.6642 (11)	0.0818 (8)	0.084 (5)	
H53A	0.2270	0.7128	0.1157	0.126*	
H53B	0.2356	0.6104	0.0892	0.126*	
H53C	0.1684	0.6477	0.0911	0.126*	
C54	0.2017 (6)	0.6948 (9)	0.0098 (7)	0.059 (3)	
C55	0.3156 (8)	0.4400 (11)	0.4141 (8)	0.079 (4)	
H55A	0.3164	0.4092	0.3676	0.118*	
H55B	0.3409	0.4955	0.4184	0.118*	
H55C	0.2732	0.4565	0.4152	0.118*	
C56	0.3386 (7)	0.3822 (11)	0.4716 (8)	0.057 (4)	
F1	0.5218 (5)	0.5503 (8)	0.3177 (7)	0.107 (4)	
F2	0.4542 (7)	0.6128 (10)	0.4395 (6)	0.140 (5)	
F3	0.4492 (7)	0.6520 (8)	0.3254 (6)	0.158 (6)	
F4	0.5169 (5)	0.5071 (8)	0.4329 (5)	0.095 (4)	
F5	0.5426 (7)	0.6515 (8)	0.4094 (6)	0.129 (5)	
F6	0.4350 (4)	0.5105 (8)	0.3415 (5)	0.101 (3)	
F7	0.2000 (3)	0.6676 (4)	0.5720 (3)	0.0510 (16)	
F8	0.2549 (3)	0.5503 (5)	0.5384 (4)	0.062 (2)	
F9	0.2081 (4)	0.5770 (5)	0.4199 (4)	0.068 (2)	
F10	0.1541 (4)	0.6900 (7)	0.4542 (5)	0.063 (2)	
F11	0.2579 (3)	0.6905 (5)	0.4913 (4)	0.066 (2)	
F12	0.1508 (3)	0.5525 (5)	0.5020 (5)	0.068 (2)	
F13	−0.0032 (5)	0.6054 (9)	0.1681 (5)	0.109 (4)	
F14	0.0776 (3)	0.5921 (8)	0.1137 (5)	0.079 (3)	
F15	0.0179 (4)	0.6271 (6)	0.0040 (5)	0.072 (2)	
F16	−0.0633 (3)	0.6303 (5)	0.0573 (4)	0.062 (2)	
F17	0.0169 (3)	0.7190 (5)	0.0984 (4)	0.065 (2)	
F18	−0.0041 (4)	0.5076 (5)	0.0676 (7)	0.102 (4)	
Ir1	0.25717 (3)	0.07100 (2)	0.22916 (3)	0.01852 (8)	
N1	0.1991 (5)	0.1828 (8)	0.2413 (6)	0.018 (2)	
N2	0.0540 (7)	0.5722 (7)	0.3447 (8)	0.028 (3)	
N3	−0.0202 (5)	0.6343 (8)	0.4028 (6)	0.041 (3)	
N4	0.0357 (6)	0.7290 (9)	0.3376 (8)	0.041 (3)	
N5	0.3110 (5)	0.1880 (8)	0.2146 (7)	0.024 (3)	
N6	0.4664 (6)	0.5746 (6)	0.1255 (8)	0.025 (3)	
N7	0.4839 (6)	0.7316 (8)	0.1344 (7)	0.036 (3)	
N8	0.5457 (5)	0.6371 (7)	0.0792 (6)	0.032 (2)	
N9	0.2980 (6)	0.0656 (6)	0.3415 (7)	0.020 (3)	
N10	0.2188 (6)	0.0582 (7)	0.1215 (7)	0.023 (3)	
N11	0.2633 (5)	0.5614 (6)	0.2520 (5)	0.047 (3)	
N12	0.1991 (8)	0.7186 (11)	−0.0486 (7)	0.109 (6)	
N13	0.3612 (8)	0.3294 (13)	0.5157 (10)	0.093 (6)	
P1	0.4866 (2)	0.5830 (3)	0.3762 (3)	0.0604 (11)	
P2	0.20527 (14)	0.6201 (2)	0.49735 (18)	0.0384 (7)	
P3	0.00657 (16)	0.6120 (3)	0.0852 (2)	0.0445 (8)	

### Atomic displacement parameters (Å^2^)

**Table:**

	*U*^11^	*U*^22^	*U*^33^	*U*^12^	*U*^13^	*U*^23^
C1	0.019 (4)	0.016 (5)	0.009 (5)	−0.002 (4)	0.000 (3)	0.002 (4)
C2	0.027 (5)	0.019 (5)	0.021 (6)	−0.001 (4)	0.004 (4)	−0.001 (4)
C3	0.025 (5)	0.023 (5)	0.018 (6)	−0.001 (4)	0.006 (4)	−0.001 (4)
C4	0.017 (5)	0.024 (5)	0.020 (6)	−0.003 (4)	0.003 (4)	−0.001 (4)
C5	0.023 (5)	0.015 (5)	0.032 (8)	−0.005 (4)	0.012 (5)	−0.005 (5)
C6	0.026 (7)	0.022 (5)	0.026 (7)	−0.004 (4)	0.006 (6)	−0.001 (4)
C7	0.031 (6)	0.033 (5)	0.027 (6)	0.001 (4)	0.013 (5)	0.003 (4)
C8	0.025 (6)	0.031 (6)	0.027 (5)	0.002 (4)	0.007 (4)	0.003 (4)
C9	0.025 (5)	0.031 (5)	0.032 (5)	−0.002 (4)	0.010 (4)	−0.002 (4)
C10	0.024 (5)	0.019 (5)	0.024 (5)	0.003 (4)	0.005 (4)	0.002 (4)
C11	0.018 (5)	0.029 (5)	0.025 (6)	−0.001 (3)	0.003 (4)	−0.004 (4)
C12	0.035 (7)	0.031 (5)	0.082 (10)	0.005 (4)	0.038 (7)	0.003 (5)
C13	0.043 (6)	0.028 (5)	0.050 (7)	0.000 (4)	0.020 (6)	−0.006 (5)
C14	0.042 (7)	0.021 (5)	0.080 (10)	0.009 (4)	0.031 (7)	0.015 (5)
C15	0.029 (5)	0.028 (5)	0.034 (8)	0.000 (4)	0.014 (5)	−0.001 (5)
C16	0.016 (5)	0.027 (6)	0.030 (7)	0.001 (4)	0.013 (4)	−0.001 (5)
C17	0.019 (5)	0.026 (5)	0.032 (8)	0.000 (4)	0.010 (5)	−0.001 (4)
C18	0.019 (6)	0.028 (6)	0.043 (9)	0.001 (4)	0.015 (5)	−0.001 (5)
C19	0.022 (5)	0.035 (6)	0.024 (7)	−0.003 (4)	0.005 (5)	−0.001 (5)
C20	0.022 (7)	0.026 (6)	0.034 (8)	−0.005 (4)	0.008 (6)	0.000 (4)
C21	0.044 (8)	0.029 (6)	0.085 (11)	−0.007 (5)	0.043 (8)	−0.011 (6)
C22	0.060 (10)	0.029 (7)	0.093 (13)	−0.012 (6)	0.059 (10)	−0.017 (6)
C23	0.028 (6)	0.021 (5)	0.054 (8)	0.005 (4)	0.022 (6)	0.002 (4)
C24	0.041 (8)	0.024 (6)	0.072 (10)	0.004 (5)	0.032 (7)	0.006 (5)
C25	0.018 (5)	0.027 (4)	0.024 (6)	−0.001 (3)	0.001 (4)	0.002 (4)
C26	0.047 (6)	0.020 (5)	0.052 (7)	−0.015 (4)	0.017 (5)	−0.003 (5)
C27	0.045 (7)	0.025 (5)	0.056 (8)	−0.011 (4)	0.022 (6)	0.002 (5)
C28	0.021 (5)	0.042 (5)	0.057 (8)	−0.007 (4)	0.012 (5)	0.008 (5)
C29	0.024 (5)	0.015 (5)	0.021 (4)	−0.004 (4)	0.002 (3)	0.000 (3)
C30	0.032 (6)	0.033 (6)	0.020 (5)	0.002 (4)	−0.001 (4)	−0.002 (4)
C31	0.034 (7)	0.036 (6)	0.016 (5)	0.000 (5)	0.002 (4)	0.003 (4)
C32	0.036 (7)	0.040 (7)	0.017 (6)	0.002 (6)	0.001 (5)	−0.003 (5)
C33	0.020 (6)	0.033 (7)	0.020 (5)	−0.004 (5)	0.005 (4)	0.003 (4)
C34	0.024 (6)	0.027 (5)	0.021 (5)	−0.001 (5)	0.000 (4)	−0.001 (4)
C35	0.021 (6)	0.023 (7)	0.027 (5)	−0.002 (5)	0.003 (4)	−0.003 (5)
C36	0.030 (7)	0.020 (7)	0.025 (6)	0.005 (5)	0.003 (5)	0.003 (4)
C37	0.026 (5)	0.021 (6)	0.032 (6)	0.003 (4)	0.014 (4)	0.003 (5)
C38	0.025 (5)	0.020 (6)	0.032 (5)	0.006 (4)	0.003 (4)	−0.001 (4)
C39	0.024 (5)	0.029 (5)	0.027 (5)	0.001 (4)	0.007 (4)	−0.001 (4)
C40	0.025 (5)	0.019 (5)	0.033 (5)	−0.003 (4)	0.010 (4)	0.001 (4)
C41	0.031 (6)	0.032 (6)	0.029 (5)	−0.009 (5)	0.007 (4)	0.005 (4)
C42	0.042 (7)	0.044 (7)	0.024 (6)	−0.008 (6)	0.008 (5)	−0.001 (5)
C43	0.027 (6)	0.028 (6)	0.025 (6)	0.006 (5)	0.010 (5)	0.003 (5)
C44	0.039 (8)	0.022 (6)	0.027 (6)	−0.004 (6)	0.010 (5)	0.006 (5)
C45	0.026 (6)	0.020 (6)	0.021 (6)	−0.001 (4)	0.009 (5)	0.005 (4)
C46	0.022 (7)	0.020 (7)	0.021 (6)	0.003 (5)	0.011 (5)	−0.002 (5)
C47	0.020 (6)	0.036 (9)	0.023 (7)	−0.004 (5)	0.005 (5)	0.007 (5)
C48	0.036 (7)	0.042 (9)	0.021 (6)	−0.003 (6)	0.006 (5)	0.006 (6)
C49	0.040 (7)	0.017 (6)	0.041 (7)	−0.009 (5)	0.011 (5)	0.004 (5)
C50	0.042 (7)	0.030 (7)	0.024 (6)	−0.018 (5)	0.003 (5)	−0.008 (5)
C51	0.105 (12)	0.045 (8)	0.122 (13)	−0.019 (8)	0.076 (11)	−0.025 (8)
C52	0.058 (7)	0.033 (6)	0.073 (8)	0.000 (5)	0.044 (6)	−0.001 (6)
C53	0.110 (12)	0.086 (11)	0.066 (7)	0.013 (9)	0.041 (7)	0.010 (7)
C54	0.064 (8)	0.058 (8)	0.061 (6)	−0.007 (6)	0.023 (6)	0.010 (6)
C55	0.079 (11)	0.088 (10)	0.076 (9)	0.018 (8)	0.032 (8)	0.003 (7)
C56	0.071 (10)	0.060 (8)	0.041 (7)	0.001 (8)	0.016 (7)	−0.019 (5)
F1	0.093 (7)	0.144 (10)	0.097 (9)	−0.042 (7)	0.048 (7)	−0.016 (7)
F2	0.207 (14)	0.163 (11)	0.066 (7)	0.032 (12)	0.062 (8)	0.009 (7)
F3	0.286 (17)	0.090 (8)	0.071 (7)	0.108 (10)	−0.018 (8)	0.007 (6)
F4	0.101 (7)	0.099 (8)	0.069 (6)	−0.028 (6)	−0.018 (5)	0.019 (5)
F5	0.200 (13)	0.091 (8)	0.085 (7)	−0.057 (9)	0.009 (9)	−0.017 (6)
F6	0.073 (6)	0.112 (8)	0.098 (7)	−0.013 (6)	−0.024 (5)	0.024 (6)
F7	0.038 (3)	0.067 (4)	0.046 (3)	0.011 (3)	0.005 (3)	−0.020 (3)
F8	0.058 (4)	0.057 (4)	0.060 (4)	0.024 (3)	−0.010 (3)	−0.011 (3)
F9	0.082 (5)	0.077 (5)	0.046 (4)	0.021 (4)	0.014 (4)	−0.023 (3)
F10	0.056 (5)	0.064 (5)	0.065 (5)	0.025 (4)	0.003 (4)	0.000 (4)
F11	0.043 (4)	0.068 (5)	0.096 (6)	−0.005 (3)	0.031 (4)	−0.009 (4)
F12	0.055 (5)	0.070 (5)	0.076 (5)	−0.022 (4)	0.009 (4)	−0.011 (4)
F13	0.083 (6)	0.194 (11)	0.063 (6)	0.087 (8)	0.045 (5)	0.054 (7)
F14	0.030 (4)	0.139 (9)	0.067 (5)	0.016 (4)	0.010 (4)	−0.012 (5)
F15	0.090 (6)	0.071 (5)	0.062 (5)	0.023 (5)	0.033 (4)	−0.004 (4)
F16	0.054 (4)	0.065 (5)	0.068 (5)	−0.011 (4)	0.014 (4)	−0.039 (4)
F17	0.053 (4)	0.058 (4)	0.094 (5)	−0.021 (3)	0.036 (4)	−0.041 (4)
F18	0.080 (6)	0.024 (4)	0.202 (13)	0.002 (4)	0.031 (8)	0.002 (6)
Ir1	0.01925 (12)	0.01816 (12)	0.01878 (12)	0.0000 (3)	0.00550 (8)	0.0008 (3)
N1	0.019 (4)	0.017 (5)	0.015 (5)	−0.005 (3)	−0.002 (4)	0.003 (4)
N2	0.027 (5)	0.030 (5)	0.030 (5)	0.008 (4)	0.013 (4)	0.004 (3)
N3	0.049 (6)	0.029 (5)	0.060 (7)	0.001 (4)	0.041 (5)	0.001 (4)
N4	0.030 (5)	0.034 (5)	0.062 (7)	0.002 (4)	0.021 (5)	0.004 (4)
N5	0.029 (5)	0.020 (5)	0.027 (6)	−0.005 (4)	0.013 (5)	0.003 (4)
N6	0.021 (5)	0.021 (4)	0.034 (6)	0.003 (3)	0.010 (4)	−0.002 (3)
N7	0.043 (6)	0.021 (4)	0.053 (6)	−0.006 (4)	0.028 (5)	−0.005 (4)
N8	0.029 (4)	0.030 (5)	0.042 (6)	−0.001 (3)	0.019 (4)	0.005 (4)
N9	0.018 (5)	0.020 (5)	0.020 (5)	−0.007 (3)	0.002 (4)	0.002 (4)
N10	0.027 (6)	0.019 (5)	0.024 (5)	−0.001 (4)	0.007 (4)	0.005 (4)
N11	0.054 (6)	0.036 (5)	0.056 (7)	0.004 (4)	0.026 (6)	−0.006 (4)
N12	0.136 (13)	0.121 (12)	0.063 (7)	−0.068 (11)	0.008 (7)	0.025 (7)
N13	0.078 (11)	0.098 (11)	0.095 (11)	0.003 (8)	0.000 (9)	0.015 (9)
P1	0.059 (3)	0.055 (3)	0.065 (2)	0.0109 (19)	0.010 (2)	0.0109 (19)
P2	0.0353 (17)	0.0401 (17)	0.0383 (16)	0.0025 (14)	0.0047 (13)	−0.0089 (13)
P3	0.0349 (19)	0.051 (2)	0.0489 (19)	−0.0018 (15)	0.0122 (15)	−0.0149 (17)

### Geometric parameters (Å, º)

**Table:**

C1—N1	1.375 (16)	C33—H33	0.9500
C1—C2	1.421 (18)	C34—C35	1.40 (2)
C1—C15	1.489 (9)	C34—C39	1.39 (2)
C2—C3	1.382 (17)	C35—C36	1.41 (2)
C2—H2	0.9500	C35—Ir1	2.021 (16)
C3—C4	1.392 (18)	C36—C37	1.408 (19)
C3—C6	1.452 (19)	C36—H36	0.9500
C4—C5	1.394 (17)	C37—C38	1.367 (19)
C4—H4	0.9500	C37—H37	0.9500
C5—N1	1.317 (15)	C38—C39	1.352 (18)
C5—H5	0.9500	C38—H38	0.9500
C6—C10	1.39 (2)	C39—H39	0.9500
C6—C7	1.397 (18)	C40—C41	1.370 (18)
C7—C8	1.383 (19)	C40—N10	1.418 (16)
C7—H7	0.9500	C40—C45	1.459 (17)
C8—N2	1.37 (2)	C41—C42	1.414 (19)
C8—H8	0.9500	C41—H41	0.9500
C9—N2	1.341 (16)	C42—C43	1.44 (2)
C9—C10	1.400 (17)	C42—H42	0.9500
C9—H9	0.9500	C43—C44	1.357 (19)
C10—H10	0.9500	C43—H43	0.9500
C11—N4	1.273 (17)	C44—N10	1.409 (18)
C11—N3	1.334 (14)	C44—H44	0.9500
C11—N2	1.466 (16)	C45—C50	1.396 (18)
C12—N3	1.335 (15)	C45—C46	1.42 (2)
C12—C13	1.362 (18)	C46—C47	1.39 (2)
C12—H12	0.9500	C46—Ir1	2.000 (15)
C13—C14	1.372 (18)	C47—C48	1.361 (19)
C13—H13	0.9500	C47—H47	0.9500
C14—N4	1.294 (18)	C48—C49	1.388 (19)
C14—H14	0.9500	C48—H48	0.9500
C15—N5	1.336 (18)	C49—C50	1.401 (17)
C15—C16	1.343 (19)	C49—H49	0.9500
C16—C17	1.400 (16)	C50—H50	0.9500
C16—H16	0.9500	C51—C52	1.470 (17)
C17—C18	1.392 (19)	C51—H51A	0.9800
C17—C20	1.509 (19)	C51—H51B	0.9800
C18—C19	1.347 (19)	C51—H51C	0.9800
C18—H18	0.9500	C52—N11	1.140 (13)
C19—N5	1.379 (16)	C53—C54	1.390 (18)
C19—H19	0.9500	C53—H53A	0.9800
C20—C21	1.362 (19)	C53—H53B	0.9800
C20—C24	1.42 (2)	C53—H53C	0.9800
C21—C22	1.32 (2)	C54—N12	1.132 (16)
C21—H21	0.9500	C55—C56	1.37 (2)
C22—N6	1.33 (2)	C55—H55A	0.9800
C22—H22	0.9500	C55—H55B	0.9800
C23—C24	1.330 (19)	C55—H55C	0.9800
C23—N6	1.349 (15)	C56—N13	1.16 (2)
C23—H23	0.9500	F1—P1	1.549 (12)
C24—H24	0.9500	F2—P1	1.571 (12)
C25—N8	1.256 (14)	F3—P1	1.505 (10)
C25—N7	1.331 (16)	F4—P1	1.579 (11)
C25—N6	1.453 (16)	F5—P1	1.614 (12)
C26—C27	1.336 (17)	F6—P1	1.594 (10)
C26—N7	1.402 (16)	F7—P2	1.581 (6)
C26—H26	0.9500	F8—P2	1.577 (7)
C27—C28	1.352 (19)	F9—P2	1.589 (7)
C27—H27	0.9500	F10—P2	1.611 (9)
C28—N8	1.388 (15)	F11—P2	1.582 (8)
C28—H28	0.9500	F12—P2	1.582 (8)
C29—N9	1.311 (16)	F13—P3	1.608 (9)
C29—C30	1.400 (16)	F14—P3	1.582 (8)
C29—C34	1.453 (19)	F15—P3	1.602 (9)
C30—C31	1.343 (18)	F16—P3	1.553 (8)
C30—H30	0.9500	F17—P3	1.592 (8)
C31—C32	1.33 (2)	F18—P3	1.568 (9)
C31—H31	0.9500	Ir1—N10	2.011 (14)
C32—C33	1.388 (18)	Ir1—N9	2.095 (13)
C32—H32	0.9500	Ir1—N1	2.125 (11)
C33—N9	1.280 (18)	Ir1—N5	2.139 (11)
			
N1—C1—C2	118.6 (12)	C44—C43—H43	121.5
N1—C1—C15	117.9 (14)	C42—C43—H43	121.5
C2—C1—C15	123.5 (15)	C43—C44—N10	124.9 (14)
C3—C2—C1	120.5 (12)	C43—C44—H44	117.6
C3—C2—H2	119.7	N10—C44—H44	117.6
C1—C2—H2	119.7	C50—C45—C46	123.3 (12)
C4—C3—C2	119.0 (13)	C50—C45—C40	121.2 (12)
C4—C3—C6	122.4 (12)	C46—C45—C40	115.4 (12)
C2—C3—C6	118.5 (13)	C47—C46—C45	116.3 (14)
C3—C4—C5	118.1 (12)	C47—C46—Ir1	128.7 (12)
C3—C4—H4	120.9	C45—C46—Ir1	115.0 (10)
C5—C4—H4	120.9	C46—C47—C48	120.2 (15)
N1—C5—C4	123.5 (12)	C46—C47—H47	119.9
N1—C5—H5	118.3	C48—C47—H47	119.9
C4—C5—H5	118.3	C47—C48—C49	124.6 (15)
C10—C6—C7	117.0 (14)	C47—C48—H48	117.7
C10—C6—C3	121.2 (12)	C49—C48—H48	117.7
C7—C6—C3	121.8 (16)	C48—C49—C50	117.0 (12)
C8—C7—C6	122.3 (14)	C48—C49—H49	121.5
C8—C7—H7	118.8	C50—C49—H49	121.5
C6—C7—H7	118.8	C49—C50—C45	118.6 (12)
N2—C8—C7	118.4 (13)	C49—C50—H50	120.7
N2—C8—H8	120.8	C45—C50—H50	120.7
C7—C8—H8	120.8	C52—C51—H51A	109.5
N2—C9—C10	120.8 (13)	C52—C51—H51B	109.5
N2—C9—H9	119.6	H51A—C51—H51B	109.5
C10—C9—H9	119.6	C52—C51—H51C	109.5
C6—C10—C9	120.1 (13)	H51A—C51—H51C	109.5
C6—C10—H10	119.9	H51B—C51—H51C	109.5
C9—C10—H10	119.9	N11—C52—C51	177.7 (15)
N4—C11—N3	130.1 (13)	C54—C53—H53A	109.5
N4—C11—N2	116.1 (12)	C54—C53—H53B	109.5
N3—C11—N2	113.6 (12)	H53A—C53—H53B	109.5
N3—C12—C13	125.7 (13)	C54—C53—H53C	109.5
N3—C12—H12	117.2	H53A—C53—H53C	109.5
C13—C12—H12	117.2	H53B—C53—H53C	109.5
C12—C13—C14	113.3 (14)	N12—C54—C53	176.4 (18)
C12—C13—H13	123.4	C56—C55—H55A	109.5
C14—C13—H13	123.4	C56—C55—H55B	109.5
N4—C14—C13	124.4 (14)	H55A—C55—H55B	109.5
N4—C14—H14	117.8	C56—C55—H55C	109.5
C13—C14—H14	117.8	H55A—C55—H55C	109.5
N5—C15—C16	123.5 (14)	H55B—C55—H55C	109.5
N5—C15—C1	112.1 (15)	N13—C56—C55	173.4 (18)
C16—C15—C1	124.4 (16)	C46—Ir1—N10	80.8 (5)
C15—C16—C17	119.4 (13)	C46—Ir1—C35	86.0 (3)
C15—C16—H16	120.3	N10—Ir1—C35	94.2 (6)
C17—C16—H16	120.3	C46—Ir1—N9	93.9 (5)
C16—C17—C18	117.7 (13)	N10—Ir1—N9	172.5 (2)
C16—C17—C20	123.3 (13)	C35—Ir1—N9	80.1 (6)
C18—C17—C20	119.0 (12)	C46—Ir1—N1	98.4 (6)
C19—C18—C17	119.9 (13)	N10—Ir1—N1	92.3 (5)
C19—C18—H18	120.0	C35—Ir1—N1	172.7 (6)
C17—C18—H18	120.0	N9—Ir1—N1	93.8 (4)
C18—C19—N5	121.9 (14)	C46—Ir1—N5	173.1 (6)
C18—C19—H19	119.1	N10—Ir1—N5	94.4 (5)
N5—C19—H19	119.1	C35—Ir1—N5	99.4 (6)
C21—C20—C24	116.8 (14)	N9—Ir1—N5	91.3 (4)
C21—C20—C17	121.7 (16)	N1—Ir1—N5	76.6 (2)
C24—C20—C17	121.5 (13)	C5—N1—C1	120.2 (12)
C22—C21—C20	119.9 (16)	C5—N1—Ir1	124.8 (9)
C22—C21—H21	120.0	C1—N1—Ir1	114.1 (8)
C20—C21—H21	120.0	C9—N2—C8	121.2 (12)
C21—C22—N6	123.4 (15)	C9—N2—C11	120.4 (13)
C21—C22—H22	118.3	C8—N2—C11	118.5 (11)
N6—C22—H22	118.3	C12—N3—C11	111.1 (11)
C24—C23—N6	120.5 (14)	C11—N4—C14	115.3 (13)
C24—C23—H23	119.7	C15—N5—C19	117.5 (12)
N6—C23—H23	119.7	C15—N5—Ir1	118.5 (10)
C23—C24—C20	120.4 (14)	C19—N5—Ir1	123.8 (10)
C23—C24—H24	119.8	C22—N6—C23	118.7 (12)
C20—C24—H24	119.8	C22—N6—C25	122.1 (11)
N8—C25—N7	129.5 (12)	C23—N6—C25	119.2 (13)
N8—C25—N6	117.2 (12)	C25—N7—C26	113.6 (12)
N7—C25—N6	113.3 (11)	C25—N8—C28	116.6 (12)
C27—C26—N7	120.5 (14)	C33—N9—C29	122.0 (13)
C27—C26—H26	119.7	C33—N9—Ir1	124.1 (10)
N7—C26—H26	119.7	C29—N9—Ir1	113.8 (10)
C26—C27—C28	120.3 (14)	C44—N10—C40	117.2 (12)
C26—C27—H27	119.8	C44—N10—Ir1	126.2 (10)
C28—C27—H27	119.8	C40—N10—Ir1	116.6 (9)
C27—C28—N8	119.3 (12)	F3—P1—F1	93.4 (8)
C27—C28—H28	120.4	F3—P1—F2	90.3 (8)
N8—C28—H28	120.4	F1—P1—F2	176.2 (8)
N9—C29—C30	118.8 (13)	F3—P1—F4	171.8 (9)
N9—C29—C34	116.9 (12)	F1—P1—F4	92.6 (7)
C30—C29—C34	124.2 (12)	F2—P1—F4	83.8 (7)
C31—C30—C29	120.2 (13)	F3—P1—F6	86.4 (7)
C31—C30—H30	119.9	F1—P1—F6	86.5 (6)
C29—C30—H30	119.9	F2—P1—F6	94.7 (8)
C32—C31—C30	118.3 (13)	F4—P1—F6	88.4 (5)
C32—C31—H31	120.8	F3—P1—F5	95.9 (8)
C30—C31—H31	120.8	F1—P1—F5	89.7 (7)
C31—C32—C33	120.3 (15)	F2—P1—F5	88.9 (8)
C31—C32—H32	119.9	F4—P1—F5	89.7 (6)
C33—C32—H32	119.9	F6—P1—F5	175.7 (7)
N9—C33—C32	120.4 (14)	F8—P2—F12	91.9 (4)
N9—C33—H33	119.8	F8—P2—F7	92.0 (4)
C32—C33—H33	119.8	F12—P2—F7	91.3 (4)
C35—C34—C39	118.3 (14)	F8—P2—F11	90.2 (4)
C35—C34—C29	115.3 (13)	F12—P2—F11	177.9 (5)
C39—C34—C29	126.4 (13)	F7—P2—F11	88.5 (4)
C34—C35—C36	118.8 (15)	F8—P2—F9	91.3 (4)
C34—C35—Ir1	113.8 (12)	F12—P2—F9	89.0 (5)
C36—C35—Ir1	127.4 (12)	F7—P2—F9	176.7 (4)
C37—C36—C35	121.5 (16)	F11—P2—F9	91.0 (4)
C37—C36—H36	119.2	F8—P2—F10	178.9 (5)
C35—C36—H36	119.2	F12—P2—F10	87.5 (5)
C38—C37—C36	117.0 (14)	F7—P2—F10	89.0 (4)
C38—C37—H37	121.5	F11—P2—F10	90.4 (5)
C36—C37—H37	121.5	F9—P2—F10	87.7 (5)
C39—C38—C37	122.5 (13)	F16—P3—F18	90.1 (5)
C39—C38—H38	118.7	F16—P3—F14	179.3 (6)
C37—C38—H38	118.7	F18—P3—F14	89.2 (6)
C38—C39—C34	121.8 (13)	F16—P3—F17	89.0 (4)
C38—C39—H39	119.1	F18—P3—F17	176.9 (6)
C34—C39—H39	119.1	F14—P3—F17	91.7 (5)
C41—C40—N10	120.1 (12)	F16—P3—F15	90.6 (5)
C41—C40—C45	127.9 (12)	F18—P3—F15	89.1 (6)
N10—C40—C45	112.0 (12)	F14—P3—F15	89.6 (5)
C40—C41—C42	121.6 (13)	F17—P3—F15	87.9 (5)
C40—C41—H41	119.2	F16—P3—F13	89.9 (5)
C42—C41—H41	119.2	F18—P3—F13	95.6 (7)
C41—C42—C43	119.1 (13)	F14—P3—F13	90.0 (5)
C41—C42—H42	120.4	F17—P3—F13	87.4 (6)
C43—C42—H42	120.4	F15—P3—F13	175.3 (7)
C44—C43—C42	117.1 (14)		
			
N1—C1—C2—C3	2.6 (18)	C41—C40—C45—C50	1 (2)
C15—C1—C2—C3	−176.6 (10)	N10—C40—C45—C50	−180.0 (13)
C1—C2—C3—C4	−2.1 (19)	C41—C40—C45—C46	177.1 (14)
C1—C2—C3—C6	175.2 (13)	N10—C40—C45—C46	−3.4 (18)
C2—C3—C4—C5	−0.2 (19)	C50—C45—C46—C47	0 (2)
C6—C3—C4—C5	−177.3 (14)	C40—C45—C46—C47	−176.5 (13)
C3—C4—C5—N1	2 (2)	C50—C45—C46—Ir1	178.8 (12)
C4—C3—C6—C10	28 (2)	C40—C45—C46—Ir1	2.3 (17)
C2—C3—C6—C10	−149.1 (15)	C45—C46—C47—C48	−2 (2)
C4—C3—C6—C7	−149.6 (14)	Ir1—C46—C47—C48	179.1 (12)
C2—C3—C6—C7	33 (2)	C46—C47—C48—C49	3 (2)
C10—C6—C7—C8	−2 (2)	C47—C48—C49—C50	−1 (2)
C3—C6—C7—C8	176.1 (14)	C48—C49—C50—C45	−2 (2)
C6—C7—C8—N2	1 (2)	C46—C45—C50—C49	2 (2)
C7—C6—C10—C9	1 (2)	C40—C45—C50—C49	178.3 (13)
C3—C6—C10—C9	−176.9 (14)	C4—C5—N1—C1	−2 (2)
N2—C9—C10—C6	1 (2)	C4—C5—N1—Ir1	167.1 (10)
N3—C12—C13—C14	−3 (2)	C2—C1—N1—C5	−0.7 (18)
C12—C13—C14—N4	3 (3)	C15—C1—N1—C5	178.5 (10)
N1—C1—C15—N5	−9.7 (9)	C2—C1—N1—Ir1	−170.6 (9)
C2—C1—C15—N5	169.5 (15)	C15—C1—N1—Ir1	8.7 (10)
N1—C1—C15—C16	167.5 (16)	C10—C9—N2—C8	−2 (2)
C2—C1—C15—C16	−13.3 (11)	C10—C9—N2—C11	177.7 (13)
N5—C15—C16—C17	−1 (2)	C7—C8—N2—C9	1 (2)
C1—C15—C16—C17	−177.7 (10)	C7—C8—N2—C11	−178.5 (12)
C15—C16—C17—C18	−3 (2)	N4—C11—N2—C9	171.0 (14)
C15—C16—C17—C20	180.0 (15)	N3—C11—N2—C9	−12.9 (19)
C16—C17—C18—C19	4 (2)	N4—C11—N2—C8	−10 (2)
C20—C17—C18—C19	−179.1 (14)	N3—C11—N2—C8	166.6 (13)
C17—C18—C19—N5	−1 (2)	C13—C12—N3—C11	3 (2)
C16—C17—C20—C21	22 (3)	N4—C11—N3—C12	−3 (2)
C18—C17—C20—C21	−154.8 (16)	N2—C11—N3—C12	−178.8 (12)
C16—C17—C20—C24	−157.4 (17)	N3—C11—N4—C14	3 (2)
C18—C17—C20—C24	26 (2)	N2—C11—N4—C14	178.6 (14)
C24—C20—C21—C22	−3 (3)	C13—C14—N4—C11	−3 (3)
C17—C20—C21—C22	177.5 (18)	C16—C15—N5—C19	4 (2)
C20—C21—C22—N6	−1 (3)	C1—C15—N5—C19	−179.1 (9)
N6—C23—C24—C20	1 (3)	C16—C15—N5—Ir1	−171.3 (12)
C21—C20—C24—C23	3 (3)	C1—C15—N5—Ir1	6.0 (13)
C17—C20—C24—C23	−177.2 (15)	C18—C19—N5—C15	−3 (2)
N7—C26—C27—C28	−4 (2)	C18—C19—N5—Ir1	171.8 (11)
C26—C27—C28—N8	6 (2)	C21—C22—N6—C23	6 (3)
N9—C29—C30—C31	−1 (2)	C21—C22—N6—C25	−176.5 (18)
C34—C29—C30—C31	−179.4 (14)	C24—C23—N6—C22	−5 (2)
C29—C30—C31—C32	−1 (2)	C24—C23—N6—C25	176.9 (14)
C30—C31—C32—C33	2 (3)	N8—C25—N6—C22	174.8 (17)
C31—C32—C33—N9	−1 (2)	N7—C25—N6—C22	−5 (2)
N9—C29—C34—C35	−3 (2)	N8—C25—N6—C23	−7.3 (19)
C30—C29—C34—C35	175.5 (13)	N7—C25—N6—C23	172.4 (13)
N9—C29—C34—C39	177.9 (14)	N8—C25—N7—C26	2 (2)
C30—C29—C34—C39	−4 (2)	N6—C25—N7—C26	−177.5 (12)
C39—C34—C35—C36	1 (2)	C27—C26—N7—C25	0 (2)
C29—C34—C35—C36	−178.2 (13)	N7—C25—N8—C28	0 (2)
C39—C34—C35—Ir1	−178.7 (12)	N6—C25—N8—C28	179.7 (12)
C29—C34—C35—Ir1	2.1 (18)	C27—C28—N8—C25	−4.2 (19)
C34—C35—C36—C37	−1 (2)	C32—C33—N9—C29	0 (2)
Ir1—C35—C36—C37	179.0 (11)	C32—C33—N9—Ir1	177.0 (11)
C35—C36—C37—C38	−1 (2)	C30—C29—N9—C33	1 (2)
C36—C37—C38—C39	3 (2)	C34—C29—N9—C33	179.8 (13)
C37—C38—C39—C34	−3 (2)	C30—C29—N9—Ir1	−176.2 (10)
C35—C34—C39—C38	1 (2)	C34—C29—N9—Ir1	2.4 (16)
C29—C34—C39—C38	179.8 (13)	C43—C44—N10—C40	−2 (2)
N10—C40—C41—C42	−2 (2)	C43—C44—N10—Ir1	177.9 (12)
C45—C40—C41—C42	177.4 (15)	C41—C40—N10—C44	2 (2)
C40—C41—C42—C43	1 (2)	C45—C40—N10—C44	−177.3 (12)
C41—C42—C43—C44	−1 (2)	C41—C40—N10—Ir1	−177.4 (10)
C42—C43—C44—N10	1 (2)	C45—C40—N10—Ir1	3.0 (15)
